# Prevalence of Psychotropic Drug Treatment Interventions in Patients Undergoing Metabolic and Bariatric Surgery: A Retrospective Study

**DOI:** 10.1007/s11695-026-08731-0

**Published:** 2026-07-15

**Authors:** Susannah Anna-Maria Leilani Wever, Dionne Sizoo, Eric Nico van Roon, Victoria Hiu Yeung Cheung, Michiel Adriaan Damhof, Marloes Emous, Berdine Emma Oortgiesen

**Affiliations:** 1 Unit of Pharmacotherapy, Epidemiology and Economics, Department of Pharmacy, Faculty of Science and Engineering, University of Groningen, Groningen, Netherlands; 2 Department of Clinical Pharmacy and Pharmacology, Frisius Medical Centre, Leeuwarden, Netherlands; 3 Center for Obesity Northern-Netherlands (CON), Department of Bariatric and Metabolic Surgery, Frisius Medical Centre, Leeuwarden, Netherlands; 4 Department of Hospital Psychiatry, Frisius Medical Centre, Leeuwarden, Netherlands

## Abstract

**Background:**

The pharmacokinetics of oral psychotropic drugs can change after metabolic and bariatric surgery (MBS), affecting drug exposure. Given the risk of psychiatric decompensation, patients using psychotropic medications constitute a high-risk group requiring careful post-MBS monitoring. In particular, lithium, tricyclic antidepressants (TCAs), and clozapine are of concern because of their well-established dose–effect relationships, susceptibility to MBS-related exposure changes, and suitability for therapeutic drug monitoring (TDM). This study aimed to examine the prevalence of treatment interventions related to these medications in bariatric patients to prevent drug-related problems such as under- or overexposure requiring dosage adjustments or drug discontinuation.

**Methods:**

In this retrospective study, patient records from January 2017 to December 2023 were reviewed. Patients who underwent MBS and were using lithium, TCAs (amitriptyline, clomipramine, dosulepin, doxepin, imipramine, maprotiline, nortriptyline), and/or clozapine were included. Baseline characteristics, treatment interventions, and available drug plasma concentrations were collected preoperatively and up to one year postoperatively.

**Results:**

A total of 163 patients were included. Psychotropic drug adjustments were observed in 27 patients (16.6%) within the first year following MBS. Both pre- and postoperative drug plasma concentrations were available for three patients (1.8%). Three patients experienced worsening of psychiatric symptoms that necessitated hospitalization or intensive monitoring. No statistically significant differences in intervention rates were observed among patients with psychiatric disorders or pain.

**Conclusion:**

Although only a minority of patients required psychotropic drug treatment interventions, monitoring psychotropic drug treatment is essential for safe and effective treatment post-MBS. Greater attention is needed regarding altered drug exposure, and plasma concentration monitoring may help optimize psychotropic pharmacotherapy in these patients.

**Graphical Abstract:**

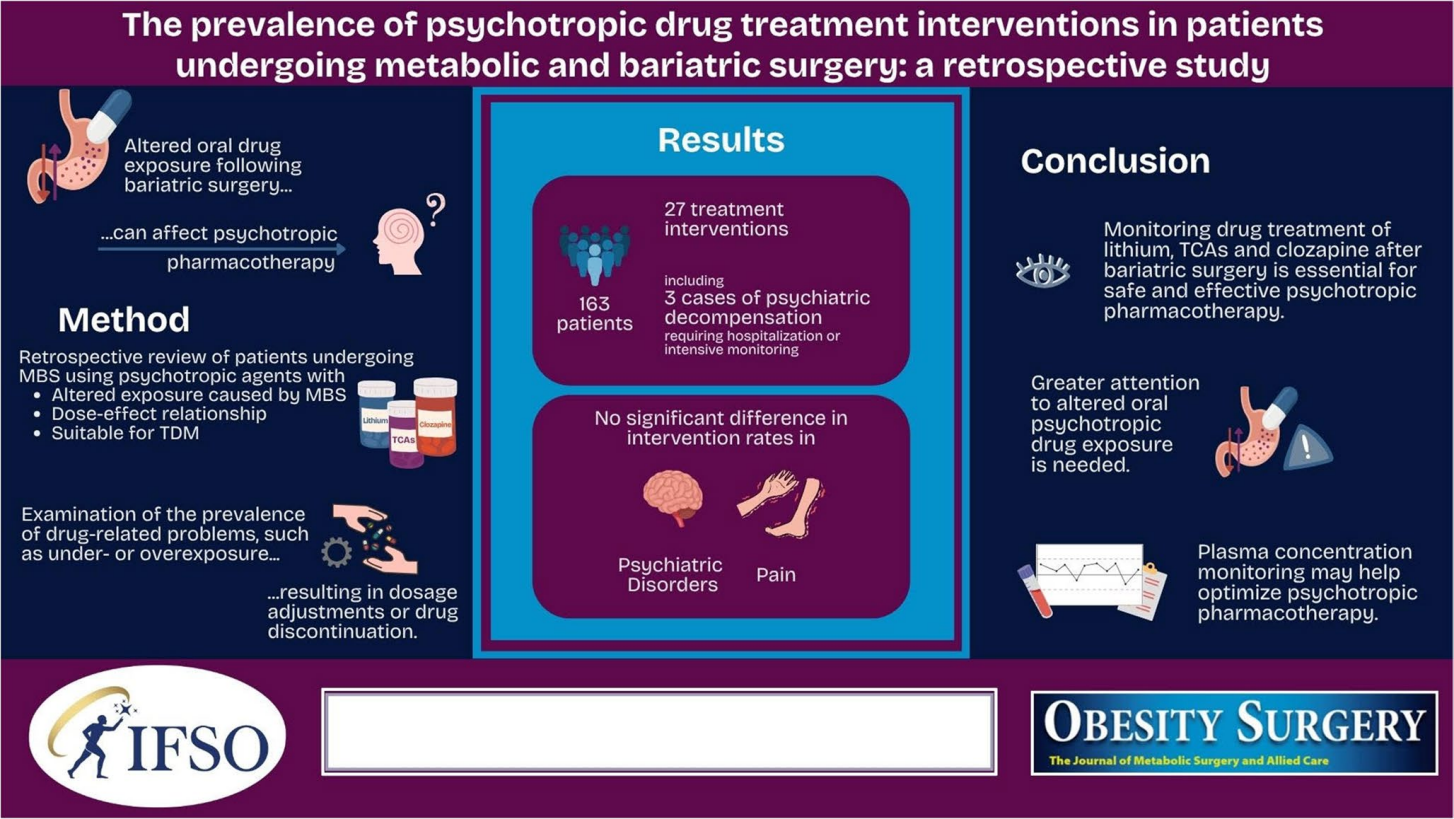

## Key Points

MBS alters the pharmacokinetics of oral psychotropics, requiring close monitoring.

Psychotherapy interventions occurred in 16.6% of patients within one year post-MBS.

Clozapine users showed highest intervention risk, in contrast to lithium users.

Plasma drug concentrations may support dosing post-MBS but need further evaluation.

## Introduction

In metabolic and bariatric surgery (MBS), anatomical alterations in the gastrointestinal tract influence the pharmacokinetics of oral drugs, thereby affecting systemic drug exposure. These pharmacokinetic changes pose a challenge for therapeutic drug management (TDM) [1, 2], particularly because the awareness and knowledge of MBS‑related pharmacokinetic alterations among prescribers remain insufficient [3]. Patients using psychotropic medications constitute a high-risk group, as they are vulnerable to psychiatric decompensation, necessitating careful perioperative monitoring [4–7]. Furthermore, among patients who undergo metabolic and bariatric surgery, the use of psychotropic drugs is frequently continued postoperatively [8–10]. Given that postoperative pharmacokinetic changes are plausible and clinically meaningful, close monitoring is essential to prevent loss of symptom control, especially in this vulnerable patient group, in whom inadequate exposure or abrupt pharmacokinetic shifts may precipitate psychiatric destabilization [2, 11–14].

Despite these concerns, the current understanding of changes in psychotropic drug exposure following metabolic and bariatric surgery is limited [3]. The absence of standardized monitoring practices impedes progress in understanding these effects and improving psychotropic drug therapy.

Studies have shown that the area under the curve (AUC) for lithium following MBS has been increased [15, 16]. Given the narrow therapeutic window of lithium, even modest changes in exposure may have clinical consequences; however, predicting pharmacokinetic shifts in individuals remains challenging [1, 2, 17]. With respect to tricyclic antidepressants (TCAs), postoperative decreases in drug plasma concentrations have been observed, although the associated clinical effects remain unclear due to substantial inter- and intraindividual variability [2]. Clozapine exposure likewise exhibits considerable variability, warranting heightened monitoring after MBS [18]. These medications have well-defined dose–effect relationships that may be altered by MBS. Moreover, TDM is available for these agents, thereby supporting appropriate dosing in clinical practice [2].

Research on the impact of MBS on psychotropic drug therapy has focused primarily on pharmacokinetic endpoints, with limited attention to clinical outcomes such as treatment interventions or psychiatric decompensation. While these studies offer valuable insights into potential alterations in drug absorption and bioavailability, they do not fully elucidate the implications for practical pharmaceutical treatment. Integrating pharmacokinetic knowledge with real-world clinical treatment outcomes may help translate these findings into clinical practice improvements to optimize psychotropic drug therapy following MBS.

The aim of this study was to determine the prevalence of psychotropic drug-related interventions in patients undergoing MBS. Furthermore, this study evaluates clinical outcomes to demonstrate the importance of individual monitoring of psychotropic drug treatments following MBS.

## Methods

### Study Design and Participants

This retrospective study was conducted between January 2017 and December 2023. Patients who underwent MBS and were using lithium, TCAs (amitriptyline, clomipramine, dosulepin, doxepin, imipramine, maprotiline, nortriptyline), and/or clozapine were included. Psychotropic medication use was identified through preoperative anesthetic screening, which consisted of a structured patient interview and verification by a pharmacy assistant using the National Medication Record. In addition, postoperative follow‑up visits were reviewed to confirm continued medication use. For a small number of patients, laboratory confirmation was available.

In this study, psychotropic medications were selected for which pharmacokinetic changes after MBS were likely to be clinically relevant and for which TDM is considered as the standard of care according to current clinical guidelines. Medication selection was therefore based on three predefined criteria: (1) a known dose–effect relationship with predefined reference values to identify potential under‑ or overtreatment, (2) an expected influence of MBS on pharmacokinetics, especially drug absorption, based on available evidence, and (3) the availability of a validated drug assay. The narrow therapeutic range of lithium provided an additional rationale for inclusion [2]. In contrast, for other commonly used psychotropic medications, such as selective serotonin reuptake inhibitors, TDM is not routinely used to guide dosing, as treatment decisions are guided primarily by clinical presentation rather than plasma concentrations [2]. Medication selection was established in consultation with psychiatrists, surgeons, and hospital pharmacists.

Exclusion criteria included those patients who had undergone ring-augmented procedures, those who had undergone a redo and/or conversion within one year after the primary surgery, and those who had received enteral (tube) feeding within one year after surgery.

### Data Collection

Baseline characteristics, including sex, age, height, weight, surgical technique, indication for psychotropic medication, preoperative drug plasma concentrations, daily dosage, and medication use, were obtained from the electronic patient records (EPRs). Postoperative drug plasma concentration(s), daily dosage, medication use, and treatment interventions were collected at predefined postoperative time points during the first year after MBS, and the underlying reason for each intervention was also recorded.

### Outcome Measures

The primary outcome measure was the occurrence of psychotropic drug adjustments within one year after MBS, defined as changes in daily dosage, drug discontinuation, altered use, or other medication-related adjustments. Secondary outcome measures included the occurrence of drug plasma concentration determinations, plasma concentrations relative to the therapeutic range, and differences between pre- and postoperative drug plasma concentrations.

### Statistical Analysis

Descriptive analyses were performed using SPSS Statistics version 28. Categorical variables are presented as counts and percentages. Quantitative variables are reported as the mean ± SD when normally distributed and as the median (IQR) when non-normally distributed, on the basis of the assessments using histograms, Q–Q plots, and the Shapiro–Wilk test. Categorical variables were analyzed using the chi-square test when expected cell counts were sufficient and Fisher’s exact test when expected counts were small (< 5). A probability value of *p* < 0.05 was considered to indicate statistical significance.

### Ethical Considerations

The study protocol was reviewed by the institutional research review committee, which confirmed that it met applicable ethical and legal standards for non‑interventional medical research. The committee determined that the Medical Research Involving Human Subjects Act did not apply to this study.

## Results

**Fig. 1:**
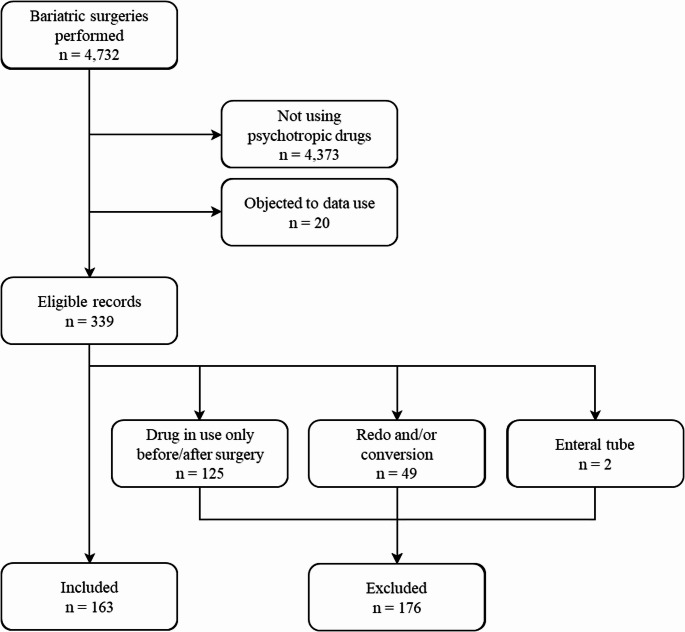
Flow diagram based on the inclusion and exclusion criteria

During the study period, 4,732 patients underwent MBS, of whom 163 (3.4%) were included (Fig. 1). The cohort consisted of 149 women and 14 men, with a median age of 52 years (IQR 16) and a median BMI at the time of surgery of 41.5 kg/m^2^ (IQR 7.0). No patients used dosulepin, doxepin, or maprotiline during the study period. Baseline characteristics are presented in Table 1.

**Table 1:** Baseline characteristics of the study population

Baseline Characteristic	*N* = 163
Sex, female; n (%)	149 (91.4)
Age on the day of surgery, years; median (IQR)	52 (16)
BMI on the day of surgery, kg/m^2^; median (IQR)	41.5 (7.0)
Surgical technique	
Gastric sleeve, n (%)	10 (6.1)
Roux-and-Y gastric bypass, n (%)	64 (39.3)
One-Anastomosis gastric bypass, n (%)	89 (54.6)
Indication^a^	
Psychiatric disorder, n (%)	70 (42.9)
Pain, n (%)	102 (62.6)
Irritable bowel syndrome, n (%)	3 (1.8)
Psychotropic drug in usage^a^	
Lithium carbonate, n (%)	3 (1.8)
TCA, n (%)	
Amitriptyline, n (%)	129 (79.1)
Clomipramine, n (%)	13 (8.0)
Nortriptyline, n (%)	16 (9.8)
Other TCAs, n (%)	1 (0.6)
Clozapine, n (%)	2 (1.2)

*n* number *IQR* interquartile range, *BMI* body mass index, *TCA* tricyclic antidepressant. ^a^Patients may have been counted more than once

Overall, 27 patients (16.6%) required postoperative psychotropic drug treatment interventions. Intervention rates did not differ significantly between patients treated for psychiatric disorders or those treated for pain. TCAs were prescribed to 66 patients for psychiatric indications and to 105 patients for pain or IBS (Table 2).

**Table 2:** Prevalence of psychotropic drug treatment interventions (*n* = 163)

	LI	AMI	CLOM	NOR	IMI	CLOZ	Total	Intervention *n* (%)	No intervention, *n* (%)	*p*
Psychiatric disorder^a^	3	46	13	7	0	2	70^a^	13 (18.6)	57 (81.4)	0.671
Pain	0	91	1	9	1	0	102	17 (16.7)	85 (83.3)	1.000
IBS	0	3	0	0	0	0	3	1 (33.3)	2 (66.7)	n.a.
							**Total**	27 (16.6)	136 (83.4)	

*LI* lithium, *AMI* amitriptyline, *CLOM* clomipramine, *NOR* nortriptyline, *IMI* imipramine, *CLOZ* clozapine, *IBS* irritable bowel syndrome. ^a^Patients may have been counted more than once

As shown in Table 3, all patients using clozapine (*n* = 2) required an intervention, whereas no interventions occurred among patients using lithium or imipramine (Table 3). Most interventions (18.5%) were based on clinical presentation and primarily involved patients using TCAs. Interventions guided solely by plasma concentrations accounted for 3.7%, and another 3.7% were based on both clinical symptoms and plasma concentrations. In 74.1% of cases, the reason for intervention could not be retrieved.

**Table 3:** Reasons for treatment intervention (*n* = 163)

Drug	Intervention, *n* (%)	Clinical presentation, *n* (%)	Plasma concentration, *n* (%)	Plasma concentration and clinical presentation, *n* (%)	Unknown, *n* (%)
LI	0 (0.0)	n.a.	n.a.	n.a.	n.a.
AMI	19 (14.7)	3 (15.8)	0 (0.0)	0 (0.0)	16 (84.2)
CLOM	2 (15.4)	1 (50.0)	1 (50.0)	0 (0.0)	0 (0.0)
NOR	4 (25.0)	1 (25.0)	0 (0.0)	0 (0.0)	3 (75.0)
IMI	0 (0.0)	n.a.	n.a.	n.a.	n.a.
CLOZ	2 (100.0)	0 (0.0)	0 (0.0)	1 (50.0)	1 (50.0)
Total	27 (16.6)	5 (18.5)	1 (3.7)	1 (3.7)	20 (74.1)

*LI* lithium, *AMI* amitriptyline, *CLOM* clomipramine, *NOR* nortriptyline, *IMI* imipramine, *CLOZ* clozapine

Preoperative drug plasma concentrations were documented in three patients (1.8%), and postoperative concentrations in five patients (3.1%), of whom three had both measurements (1.8%). In one patient using lithium, preoperative subtherapeutic drug plasma concentrations increased to therapeutic levels after surgery. Conversely, one patient using clomipramine and one using clozapine showed postoperative decreases from therapeutic to subtherapeutic concentrations. Details are shown in Table 4.

**Table 4:** Pre- and postoperative drug plasma concentration determinations (*n* = 163)

Drug	Preoperative drug plasma concentrations, *n* (%)	Outside therapeutic range, *n* (%)	Postoperative drug plasma concentrations, *n* (%)	Outside therapeutic range, *n* (%)	∆ pre/post operative
LI	1 (33.3)	1 (100.0)	2 (66.7)	0 (0.0)	+ 0.31 mmol/L
CLOM	1 (7.7)	0 (0.0)	2 (15.4)	1 (50.0)	−33 µg/L^a^
CLOZ	1 (50.0)	0 (0.0)	1 (50.0)	1 (100.0)	−153 µg/L^b^
Other TCAs	n.a.	n.a.	n.a.	n.a.	n.a.
Total	3 (1.8)	1 (33.3)	5 (3.1)	2 (40.0)	3 (1.8)

^a^ Plasma concentration outside the therapeutic range

^b^ The largest delta between pre- and postoperative measurements was recorded

Two patients experienced worsening psychiatric symptoms within one year after MBS, in which one required hospitalization. In both cases, subtherapeutic drug plasma concentrations were identified and subsequently guided treatment adjustments. The interval to intervention differed markedly between these patients, occurring either early postoperatively or several months after surgery. Across the full cohort, the timing of treatment adjustments varied considerably, with interventions occurring throughout the follow‑up period.

## Discussion

This retrospective study investigated the prevalence of treatment interventions involving lithium, TCAs, and clozapine in patients who underwent MBS. The study found that 16.6% of patients required intervention within one year after surgery.

Among patients undergoing MBS, only a small proportion used the psychotropic drugs studied [10]. Furthermore, a substantial proportion of patients used these psychotropic drugs for pain, a condition in which alterations in drug plasma concentrations generally pose fewer clinical risks. In contrast, in patients using these medications for psychiatric disorders, individual cases underscore the importance of close monitoring due to the increased clinical risk associated with fluctuations in drug levels.

Notably, no lithium-related interventions were recorded within one year following MBS, although the number of patients using lithium was limited. Given lithium’s narrow therapeutic window and evidence of MBS-related changes in drug plasma concentrations [1, 2], a greater need for interventions was anticipated. Case reports show varying outcomes. Ayub et al. [19] reported 12 cases of lithium toxicity following MBS, whereas Bingham et al. [20] reported variable drug plasma concentrations among patients, but as in the present study outcomes, no intervention was required in two of the three cases described. These discrepancies may reflect publication bias, underscoring the need for larger, prospective studies.

A small group of patients using TCAs required intervention, primarily based on clinical presentation. This can be attributed to the high proportion of patients using TCAs for pain, a condition in which clinical symptoms often guide management more than drug plasma levels. Although studies describe postoperative changes in TCA plasma concentrations, interventions in TCA dosing have not been specifically described in the literature [2, 13, 21]. The individual cases in this study indicate that monitoring may be particularly valuable when TCAs are used for psychiatric disorders, as evidenced by one patient who developed psychiatric decompensation. This finding suggests that preoperative plasma concentrations may serve as a useful reference point for postoperative management, in addition to clinical evaluation.

In the study cohort, all patients using clozapine required some form of treatment intervention. However, the small number of cases renders formal statistical comparisons inappropriate, and the findings are therefore presented descriptively. In two case reports by Kaltsounis and De León [22] and Mahgoub et al. [23], two patients experienced psychiatric decompensation after MBS, which is consistent with the findings in the present study. In addition, plasma drug concentrations decreased after surgery in this study. Since patients using clozapine in both the previous and present studies achieved clinical stability following intervention, this suggests that MBS affects clozapine exposure and necessitates intervention. These findings confirm the need to monitor clozapine use in MBS patients both clinically and by measuring drug plasma concentrations [22, 23].

The study outcomes demonstrate substantial interindividual variability in postoperative changes in psychotropic drug exposure. The wide distribution in time to intervention also highlights this variability and emphasizes the importance of careful monitoring of individual patients and their medications, particularly during the first postoperative year, as pharmacokinetic changes may arise early postoperatively or months after surgery.

In two of the three patients for whom pre- and postoperative drug plasma concentrations were determined, plasma monitoring directly informed treatment interventions, demonstrating their value on a small scale. Recent studies emphasize the value of drug plasma concentration determinations in addition to monitoring clinical presentation and highlight their relevance in MBS care [2, 13, 14, 21]. However, the applicability of established therapeutic ranges in post-MBS patients remains uncertain, and further research is needed to validate target concentrations in this population.

A key strength of this study is that it provides results based on real-world clinical practice, focusing on treatment interventions rather than solely on pharmacokinetic endpoints. In addition, the secondary research questions shed light on more theoretical issues with clinical implications, such as the utility of drug plasma concentrations in pharmacological monitoring. Furthermore, in collaboration with psychiatrists, surgeons, and hospital pharmacists, relevant psychotropic drugs were identified for the study design. The dose–effect relationships of the included drugs are well established, the expected impact of MBS on pharmacokinetics is supported, and validated drug assays are available. This approach supports the translation of the study findings into clinical practice, as plasma concentration monitoring is directly informative and routinely applied for these medications. However, the small population from a single center may not be representative of national psychotropic drug monitoring practices associated with MBS. For several drugs, fewer than 20, or even fewer than 5, patients were included in the patient’s group, limiting statistical power. Additionally, the retrospective design may have introduced bias in the recorded data. Many patients receive psychiatric care outside the hospital, including primary care and community mental health services, which can lead to incomplete documentation in the EPR. Therefore, conclusions regarding the indications for intervention should be interpreted with caution. However, this limitation primarily affects the ability to determine why interventions occurred and does not substantially influence the primary outcome measure, namely the prevalence of interventions. To mitigate this limitation in clinical practice, the MBS care team should proactively notify and advise primary care about patients at risk and the need for TDM both preoperatively and postoperatively. This includes providing structured guidance on when to obtain baseline levels, when to re-measure early after surgery, and when to repeat testing during long‑term follow‑up. Furthermore, psychiatric disorders often follow a relapsing and remitting course, and medication adjustments may occur as part of the illness itself, independent of MBS. Nonetheless, for the psychotropic agents studied, there is a clear rationale to consider MBS‑related pharmacokinetic risk, given their dose–response relationships, literature-documented alteration in pharmacokinetics, and narrow therapeutic windows [1, 2]. Additionally, patients are required to be clinically stable before surgery, as assessed by a multidisciplinary team that includes a psychologist and a psychiatrist. The one‑year follow‑up period is another limitation, as pharmacokinetic effects related to continued weight loss and gastrointestinal adaptation may persist beyond one year, although data on long‑term outcomes remain limited [2].

A significant gap in MBS care is the insufficient attention given to altered drug exposure after MBS in general, and to oral psychotropic drugs in particular. By demonstrating cases in which altered drug exposure was associated with clinically relevant outcomes, this study helps identify potential risks and challenges in post‑MBS pharmacotherapy. Greater awareness of these issues may enhance vigilance among health care providers.

Furthermore, the real‑world therapeutic adjustments described in this study extend existing theoretical knowledge into clinical practice. Improved awareness, combined with targeted TDM informed by both the literature and the present findings, may enhance the safety and effectiveness of psychotropic treatment after MBS and, ultimately, improve bariatric care.

## Conclusion

This study highlights the importance of closely monitoring psychotropic drug treatment in a small group of patients undergoing MBS. The potential for serious psychiatric decompensation, as demonstrated by the cases presented, underscores the necessity for vigilant observation to ensure effective and safe treatment. Drug plasma concentration determination is a valuable tool for monitoring psychotropic drug therapy, as it facilitates appropriate dosing following MBS. However, further research is needed to establish its utility on a larger scale.

## Data Availability

The data that support the findings of this study are not openly available due to reasons of sensitivity and are available from the corresponding author upon reasonable request. Data are located in controlled access data storage at Frisius Medical Centre.
